# Radial Artery Spasm—A Review on Incidence, Prevention and Treatment

**DOI:** 10.3390/diagnostics14171897

**Published:** 2024-08-29

**Authors:** Adrian Sebastian Zus, Simina Crișan, Silvia Luca, Daniel Nișulescu, Mihaela Valcovici, Oana Pătru, Mihai-Andrei Lazăr, Cristina Văcărescu, Dan Gaiță, Constantin-Tudor Luca

**Affiliations:** 1 Cardiology Department, “Victor Babes” University of Medicine and Pharmacy, 2 Eftimie Murgu Sq., 300041 Timisoara, Romania; adrian.zus@umft.ro (A.S.Z.); silvia.luca0@student.umft.ro (S.L.); oana.patru@umft.ro (O.P.); lazar.mihai@umft.ro (M.-A.L.); cristina.vacarescu@umft.ro (C.V.); dgaita@cardiologie.ro (D.G.); constantin.luca@umft.ro (C.-T.L.); 2 Institute of Cardiovascular Diseases Timisoara, 13A Gheorghe Adam Street, 300310 Timisoara, Romania; daniel.nisulescu@umft.ro; 3 Research Center of the Institute of Cardiovascular Diseases Timisoara, 13A Gheorghe Adam Street, 300310 Timisoara, Romania; 4 Department of Histology, Faculty of Medicine, Vasile Goldis Western University of Arad, 310025 Arad, Romania

**Keywords:** radial artery spasm, transradial access, coronary interventions, prevention, treatment methods, incidence, risk factors, vasodilators, catheter techniques, patient management

## Abstract

Radial artery spasm (RAS) is a common complication associated with transradial access (TRA) for coronary interventions, particularly affecting elderly patients in whom radial access is preferred due to its benefits in reducing bleeding complications, improving clinical outcomes, and lowering long-term costs. This review examines the incidence, prevention, and treatment of RAS. Methods included an online search of PubMed and other databases in early 2024, analyzing meta-analyses, reviews, studies, and case reports. RAS is characterized by a sudden narrowing of the radial artery due to psychological and mechanical factors with incidence reports varying up to 51.3%. Key risk factors include patient characteristics like female sex, age, and small body size as well as procedural factors such as emergency procedures and the use of multiple catheters. Preventive measures include using distal radial access, hydrophilic sheaths, and appropriate catheter sizes. Treatments involve the intraarterial administration of nitroglycerine and verapamil as well as mechanical methods like balloon-assisted tracking. This review underscores the need for standardizing RAS definitions and emphasizes the importance of operator experience and patient management in reducing RAS incidence and improving procedural success.

## 1. Introduction

**Figure 1:**
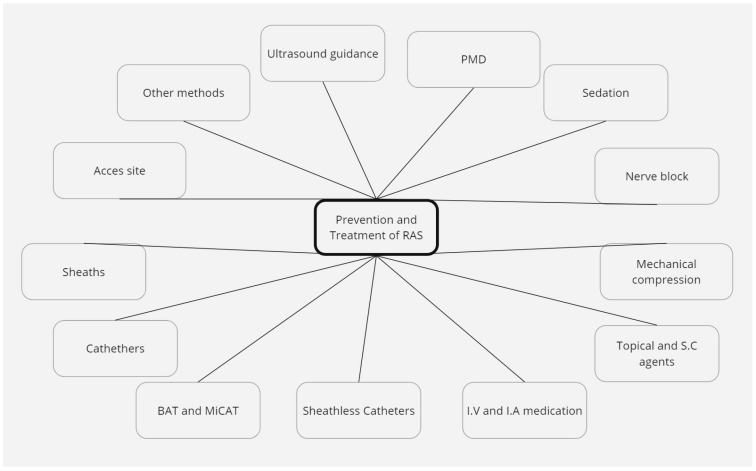
Prevention and treatment methods (I.V: intravenous, I.A: intraarterial, S.C: subcutaneous, BAT: balloon-assisted tracking, MiCAT: microcatheter-assisted tracking).

**Figure 2:**
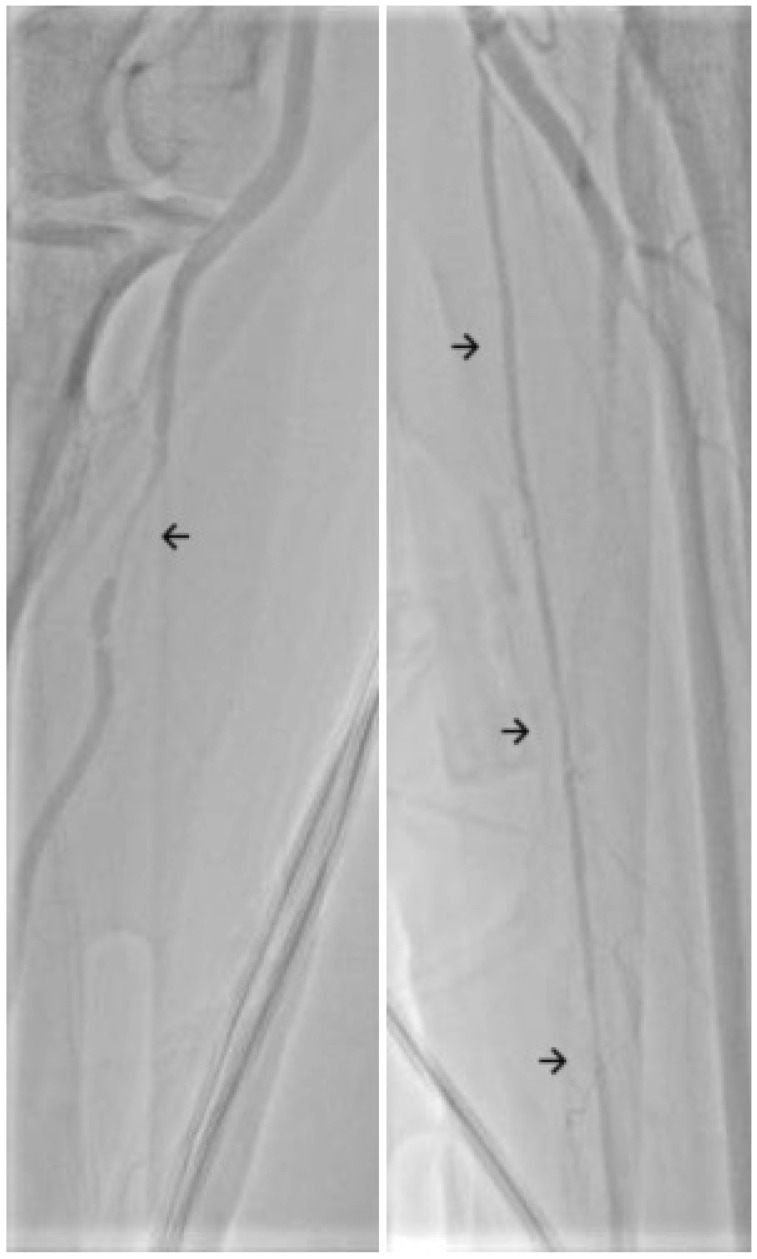
Focal (**left**) and diffuse (**right**) radial artery spasm as seen on angiography.

Radial artery spasm (RAS) is a frequent complication when using transradial access (TRA) for coronary artery interventions [1,2]. TRA is the recommended access site in both acute and chronic presentation, especially in elderly patients [3,4], due to several benefits, including less bleeding complications with improved clinical outcomes [5,6], early patient ambulation, and reduced long-term costs [7]. Spasm has a two-way relationship with pain: it can be the cause of increased discomfort for the patient, and it can be brought about by the pain related to arterial puncture and catheter and wire manipulation. As a long-term consideration, RAS leads to higher rates of radial artery occlusion (RAO) [8,9], which can rarely be symptomatic but may also compromise a future access site. From a procedural point of view, RAS increases the risk of complications, such as local hematoma formation [10], increased radiation time and dose [2,10], and requiring more frequent access site cross-over [10,11], and in extremely rare cases, it can lead to catheter entrapment [12] or arterial avulsion [13]. Thus, operators need to have a grasp of the methods used to prevent and overcome RAS (Figure 1). The definition of RAS is heterogeneous, but it is broadly considered to be a temporary and sudden narrowing of the radial artery lumen [14] due to the local release of vasoconstricting factors brought on by psychological (patient anxiety) and mechanical (local trauma caused by sheath, catheter and wire manipulation) factors [15,16]. The human radial artery is rich in alpha 1 adrenoreceptors and thus predisposed to spasm [17]. The definition of RAS is unstandardized, and it either relies on patients describing local discomfort or pain (frequently assessed using a Visual Analog Scale) and the operator sensing resistance to the manipulation of materials [18] (Table 1), or on subjective criteria, such as a >25% [19], >30% [20], >50% [21] or even >75% [22] lumen reduction on angiography (Figure 2). Alternatively, an Automated Pullback Device can be used—it retracts the sheath from within the artery after conclusion of the procedure, measuring the necessary force [23,24].

## 2. Materials and Methods

We conducted an online search of PubMed, Google Scholar, SCOPUS, Cochrane and ClinicalKey in January–February 2024, using the key phrase “radial artery spasm”, and reviewed meta-analyses, reviews, clinical studies (randomized, prospective, retrospective and observational) and case reports. Exclusion criteria were non-English articles and those that were not directly relevant to the subject.

**Figure 3:**
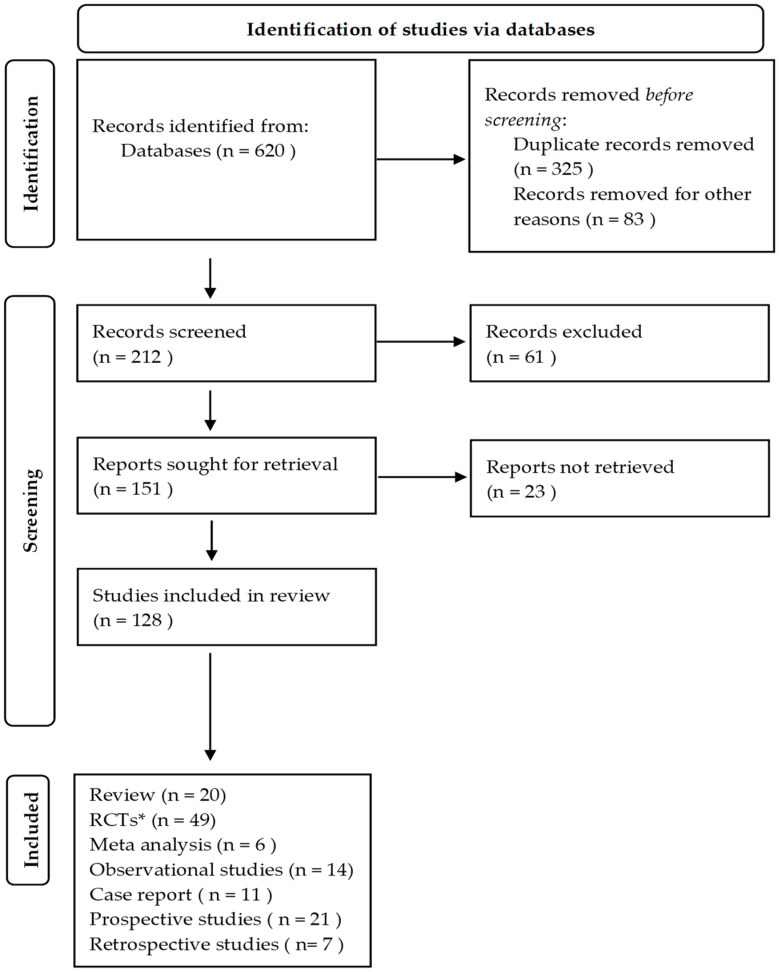
Flowchart of study selection (*RCTs: randomized control trials).

The flowchart outlines the study selection process for a systematic review shown in (Figure 3). Initially, 620 records were identified from databases with 325 duplicate records and 83 records removed for other reasons. After screening 212 records, 61 were excluded, leaving 151 reports sought for retrieval. However, 23 reports were not retrieved. Ultimately, 128 studies were included for this work, comprising 20 reviews, 49 randomized controlled trials (RCTs), 6 meta-analyses, 14 observational studies, 11 case reports, 21 prospective studies, and 7 retrospective studies. This systematic process ensures the thorough identification and inclusion of relevant studies for the review.

## 3. Results

### 3.1. Incidence and Risk Factors

Due to the heterogeneous definition and difference in local protocols on prevention, reports of incidents vary wildly, up to 51.3% (Table 2). Several predisposing factors, only some modifiable, have been documented (Table 3) [20,25]. These can be summarized into three points:

- Patient general characteristics: female sex [26,27,28], younger [29] or older [30] age, small body size [27], anxiety [27], rapid baseline heart rate [20], hypertension [31], diabetes mellitus [18], dyslipidemia [32], smoking [27], peripheral artery disease [31], history of CABG [33];
- Patient local characteristics: small radial artery size [26], low radial pulse intensity [34], anatomical variations of the radial artery (aberrant origin, loops and tortuous configuration) [22,26], dominant hand (higher RAS and RAO than nondominant hand) [35];
- Procedural characteristics: emergency procedure, multiple access attempts [32], multiple catheters used, large sheaths and catheters, long procedures [36,37], long time waiting in the catheterization laboratory [37];

Novel predictors: low heart rate variability (HRV) [38], low asymmetric dimethylarginine (ADMA) serum levels [39].

**Table 2:** Study size, criteria used for RAS diagnosis and RAS incidence according to different authors (*—no RAS reported but small study size).

Authors	Enrollment	Criteria for RAS Diagnosis	RAS Incidence (%)
Aminian et al. [40]	1307	Clinical	4
Aminian et al. [41]	114	Clinical	4.4
Aminian et al. [42]	1926	Clinical	5
Astarcioglu et al. [43]	150	Angiographic	26.6
Beyer et al. [44]	86	Clinical	25
Bochenek et al. [33]	293	Clinical	18.8
Bouchahda et al. [45]	1523	Clinical	20
Byrne et al. [46]	86	Clinical	12.9
Candemir et al. [47]	63	Clinical	16
Caussin et al. [36]	351	Clinical	11.1
Chen et al. [48]	406	Angiographic	7.3
Collet et al. [49]	220	Angiographic	9
Coppola et al. [50]	379	Clinical	11.6
Coroleu et al. [51]	736	Clinical	14.8
Costa-Mateu et al. [52]	1953	Clinical	9
Curtis et al. [29]	169	Angiographic	14.2
Dahm et al. [53]	171	Clinical	2.9
Deftereos et al. [54]	172	Clinical	7.6
Deftereos et al. [27]	2013	Angiographic	5.4
Dharma et al. [55]	150	Clinical	6
Ercan et al. [28]	81	Clinical	19.1
Ezhumalai et al. [56]	200	Clinical	4.5
Filho et al. [57]	50	Clinical	2.1
Giannopoulos et al. [31]	1582	Clinical	9.3
Gorgulus et al. [19]	1722	Angiographic	10.3
Goldsmit et al. [32]	1868	Clinical	2.7
Gopalakrishnan et al. [10]	100	Clinical	23
Gul et al. [58]	200	Clinical	6.5
Hatem et al. [38]	394	Angiographic	18.5
van der Heijden et al. [59]	165	Clinical	16
Hildick-Smith et al. [60]	500	Clinical	12
Hizoh et al. [61]	591	Clinical	1.4
Horie et al. [62]	600	Clinical	1.5
Jia et al. [20]	1427	Clinical	7.8
Khan et al. [63]	136	Clinical	13.2
Kiani et al. [64]	144	Clinical	2.7
Kiemeneij et al. [23]	100	Automated Pullback Device	15
Kiemeneij et al. [24]	50	Automated Pullback Device	8
Kim et al. [65]	150	Angiographic	51.3
Kocayiğit et al. [39]	155	Clinical	10.1
Koga et al. [66]	234	Clinical	7
Livesay et al. [67]	203	Clinical	1.4
Mikaeili Mirak et al. [68]	60	Clinical	0 *
Numasawa et al. [22]	744	Angiographic	11.2
Ouadhour et al. [69]	84	Clinical	5.9
Rathore et al. [70]	790	Clinical	29.4
Rosencher et al. [25]	731	Clinical	20.1
Ruiz-Salmeron et al. [71]	500	Clinical	18.2
Ruiz-Salmeron et al. [18]	637	Clinical	20.2
Saito et al. [72]	73	Clinical	6.8
da Silva et al. [8]	2040	Clinical	12.1
Tatlı et al. [73]	104	Clinical	18.2
Tebaldi et al. [74]	418	Angiographic	30
Toprak et al. [35]	1713	Clinical	9.6
Turan et al. [75]	101	Clinical	22
Varenne et al. [76]	1219	Clinical	10.7
Yazdi et al. [77]	120	Clinical	15
Youn et al. [21]	76	Clinical and angiographic	18.4
Zencirci et al. [34]	115	Clinical	16.5
Zencirci et al. [78]	222	Clinical	10.8

**Table 3:** Summary of RAS risk factors.

Category	Risk Factor
Patient General Characteristics	Female sex
	Younger age
	Older age Small body size Anxiety Rapid baseline heart rate Hypertension Diabetes mellitus Dyslipidemia Smoking Peripheral artery disease
Patient Local Characteristics	Small radial artery size
	Low radial pulse intensity Anatomical variations of radial artery Dominant hand (higher RAS and RAO)
Procedural Characteristics	Emergency procedure
	Multiple access attempts
	Multiple catheters used
	Large sheaths and catheters Long procedures
Novel Predictors	Low heart rate variability (HRV)
	Low asymmetric dimethylarginine ADMA levels

Predictive risk scores have been proposed with five factors to be considered: body-mass index, height, smoking status, hypertension, and peripheral artery disease (at least 4 present implies high risk of RAS, with a sensitivity of 84.5% and a specificity of 74.7%, and c-statistic of 0.945) [31], or 8: the MATRIX score (c-index of 0.71 for radial access failure) [79].

### 3.2. Prevention and Treatment Methods

#### 3.2.1. Access Site

Distal radial access (DRA) has gained ground recently, with evidence of benefits such as reduced bleeding, shorter hemostasis time, and lower rates of RAO, with high procedural success [80,81,82]. While the DISCO RADIAL trial failed to show lower RAO rates and found increased RAS with DRA use [40], a meta-analysis by Prasad et al. proved less spasm with DRA but with a higher rate of crossover to another access site [83], although the classical radial approach may still be used [84].

#### 3.2.2. Sheaths

The hydrophilic sheath proves to be less spasmogenic, while sheath length might not be a factor [36,70,85]. Alternatively, an external hydrophilic lubricant can be applied on the sheath, reducing operator-felt friction [72]. Sheath/radial artery mismatch, with a ratio >1:1, needs to be avoided, as this induces RAS [59,86]. A novel idea is that of potentially coating sheaths with NO donors, thus locally releasing the vasodilating molecule, which would hopefully reduce RAS rates [87].

#### 3.2.3. Catheters

Size and number are important when considering catheters being introduced through the radial artery. A single-catheter strategy is supported by studies [52], including a meta-analysis comparing single and dual-catheter use that showed no difference in procedural and fluoroscopy time, nor contrast volume use, but highlighted reduced RAS rates [88]. Five French (5 Fr.) guiding catheters compared to six French (6 Fr.) can lead to higher procedure success rates and lower vascular complications [53]. Hydrophilic catheters also reduce RAS compared to non-hydrophilic ones [66].

#### 3.2.4. Balloon-Assisted Tracking (BAT) and Microcatheter-Assisted Tracking (MiCAT)

Mechanical methods to overcome spasm once it has occurred can be used. Balloon-assisted tracking (BAT) is performed by using a coronary balloon catheter inflated at the tip of the guiding catheter that is having problems advancing, creating a smoother profile. This approach can lead to reduced femoral cross-over rates [89], and it has been successfully used even when perforation previously occurred [90]. BAT success is reported in all cases, with no complications, and minimal increase in procedure time [91,92,93]. Microcatheter-assisted tracking (MiCAT) also has a perfect success rate, utilizing a smaller and longer catheter (4 Fr. 125 cm Multipurpose) advanced through the lumen of the one that fails to advance [94].

#### 3.2.5. Sheathless Catheters

They can be used upfront or when there is a failure to pass standard catheters through spastic radial arteries [90]. Sheathless catheters may produce less RAS even compared to low profile slender sheaths [62]. These slender sheaths reduce RAO rates [42,70], but results on the reduction in RAS are mixed [58].

#### 3.2.6. Intravenous and Intraarterial Medication

Frequently cited methods of RAS prevention in clinical practice include 100 to 250 µg of nitroglycerine and up to 5 mg of verapamil, or a combination of both, administered into the artery after sheath placement [23,48,60,74,95,96]. A 2015 meta-analysis by Kwok et al. concluded that 5 mg of verapamil with or without nitroglycerine is the best method of preventing RAS [97]. A combination of 2.5 mg of verapamil and 1 mg of molsidomine can also provide an efficient reduction in RAS [76], and nicardipine is another calcium channel blocker that can reduce RAS when used in combination with nitrates [45]. Nicorandil seems to have similar spasmolytic effects as verapamil [65], as does magnesium sulfate [46].

RAS was not further alleviated by the addition of diltiazem or nitroprusside to intraarterial nitroglycerine [50,57], and diltiazem by itself was inferior to nitroglycerine [25], with the added side effect of an increased local burning sensation reported by patients [98]. To counter this sensation, heme has been proposed as a solution [67]. Given that alpha-1 receptors are blamed for RAS, medication to block these receptors has been tested, but phentolamine proved to be less efficient than verapamil [71]. Although sublingual nitroglycerine increases radial artery diameter [99], it is not more efficient than intraarterial administration [75]. Papaverine, a phosphodiesterase inhibitor with a spasmolytic effect, might have some advantages if used instead of nitroglycerine [77].

Heparin is often mentioned as a component of antispastic cocktails, but its use can also be attributed to the clearly beneficial effects on preventing radial artery occlusion after radial access procedures [100]. Although the routine administration of spasmolytic medication is widely used, some advocate that RAS rates are no different when deferring antispastic medication to only those cases when spasm is documented [33]. Bertrand et al. found in a 2010 survey that 14% of operators used no RAS prophylaxis [101].

#### 3.2.7. Topical and Subcutaneous Agents

Trials in surgical harvesting of the radial artery for coronary bypass grafting (CABG) identified the topical use of nitroglycerine as a viable antispastic agent [102]. The pre-angiography application of topical pharmacological agents has since been explored with nitroglycerin and lidocaine shown to locally vasodilate the radial artery without affecting systemic blood pressure [103]. A perceived advantage of this approach is the avoidance of systemic effects of spasmolytic drugs (headache and hypotension for nitroglycerine, bradycardia for verapamil), which might limit their use in patients presenting with cardiogenic shock or AV block.

A randomized trial on prophylactic nitroglycerine patches for the prevention of RAS proved no adverse effect on systemic blood pressure but did not reduce RAS [104], whereas the application of nitroglycerine gel did reduce RAS in another study [10]. Several small studies investigate the usefulness of local subcutaneous infusion of nitroglycerine at the puncture site. They all show benefits relating to time to successful arterial puncture or pulse recovery after failed puncture, with no major systemic adverse effects, and they may hint at lower rates of RAS [47,51,64,69,105].

Another approach is the use of anesthetic creams containing lidocaine, which results in less patient discomfort. While one study witnessed less RAS [73], another did not [21]. Adding nitroglycerine or verapamil to the creams did not show any benefit [44,68]. However, ethyl chloride spray, a vapocoolant used as a topical local anesthetic, did significantly reduce the occurrence of RAS [106].

#### 3.2.8. Mechanical Compression

Upper arm prolonged occlusion (10 min) using a sphygmomanometric cuff has been proven to increase the diameter of the radial artery, without changes in nitric oxide levels [107], through flow-mediated dilation (FMD) [108]. It has been demonstrated as a viable method to regain arterial pulse after puncture-induced RAS [109]. The effect on RAS is variably reported [78,110]. Prior FMD measurement can be used as a marker of RAS predisposition [54], as it investigates NO-mediated arterial dilation which can be deficient in certain groups of patients. However, endothelial dysfunction graded by EndoPAT measurements after FMD was performed failed to predict RAS [59].

#### 3.2.9. Nerve Block

Radial and/or median nerve block by the needle infusion of analgesics leads to vasodilation of the radial artery [111] through sympathetic inhibition. In practical cases, radial nerve block with 0.5% levobupivacaine has been shown to be effective in relieving cannula-induced RAS in an ICU setting [112], and brachial plexus block has been used for entrapped catheter or sheath removal from the radial and brachial artery [113,114].

#### 3.2.10. Pressure-Mediated Dilation (PMD)

High-pressure saline solution infusion through the radial sheath using an automated angiographic injection system, in patients presenting with RAS, obtained superior angiographic results compared to intraarterial nitroglycerine plus verapamil administration [49].

#### 3.2.11. Sedation

Anxiety is a normal component of the human reaction when experiencing an invasive medical intervention, especially in the setting of an acute presentation. In such situations, prior explanation of the procedure may be succinct at best, and the patient may be experiencing further distress due to their underlying medical condition. Nurses may play a key role in helping patients cope with the stress and unknowns of the procedure [115]. Anxiety can be graded using scores, such as the Hamilton Anxiety Scale, which is greater in women and predicts the occurrence of RAS [28]. Moderate sedation using midazolam, with the possible addition of fentanyl, is safe and can reduce RAS and patient discomfort in some groups [27], but it failed to show a difference in others [43].

#### 3.2.12. Ultrasound Guidance

The PRIMAFACIE-TRI trial proved that a preprocedural ultrasound scan of patients’ anatomy to gain necessary information for procedural planning yielded lower radiation time, less patient discomfort, and lower incidence of RAS, all while requiring minimal time (6.4 ± 1.8 min) [116]. Intraprocedural ultrasound-guided radial artery puncture did increase the success rate and reduce the number of attempts, but it did not lower RAS rates [117,118].

#### 3.2.13. Other Methods

The successful retrieval of entrapped catheters due to severe unrelenting RAS has been achieved by using intravenous Propofol [119] or by injection of ViperSlide [120] or Rotaglide [121] solution through the sheath and/or catheter. Warming of the upper arm has also been shown to help in cases of resistant arterial spasm [122].

### 3.3. Other Reviews and Guidelines Recommendations

Abdelazeem et al. addressed nitroglycerine administration as a preventive measure for RAS and RAO in a 2022 review and meta-analysis [123]. The 11 trials that met the inclusion criteria totaled 5814 patients, and the results were that only subcutaneous nitroglycerine proved to have the benefit of both RAS and RAO reduction, while topical and intraarterial did not.

Addressing topical medication, Curtis et al. [124] noted the sparsity and heterogeneity of existing trials and could only include three studies, which showed a reduction in RAS if topical anesthetics are used but no difference if nitroglycerine is added to the topical agent.

A 2015 pooled analysis by Kwok et al. [97] that included 22 trials concluded that 5 mg of verapamil in addition to nitroglycerine, administered intraarterially, provide the best results in RAS reduction, while also mentioning optimal sheath and catheter choice.

The American Heart Association issued a 2018 statement on radial arterial access [125], underlying the reduction in bleeding and vascular complications, especially in acute coronary syndromes, and benefits in relation to quality of life and cost reduction. Low-profile hydrophilic sheaths were noted as being preferred to minimize the risk of RAS. Mild sedation, topical lidocaine for anesthetic purposes, and a warm environment are also mentioned as useful additions to the generally accepted strategy of routine administration of intraarterial spasmolytic agents: verapamil (2.5–5 mg), nitroglycerin (100–200 μg) or nicardipine (250–500 μg) after sheath insertion, between catheter exchanges, or before sheath removal. A warning is issued in relation to using these agents in patients presenting with cardiogenic shock, severely reduced ejection fraction, or severe aortic stenosis. BAT and catheter-assisted tracking represent methods of delivering materials in the presence of established RAS. Finally, nursing goals need to be focused on providing patient comfort in order to relieve anxiety.

The 2019 Society for Cardiovascular Angiography and Interventions (SCAI) expert consensus on best practices for transradial angiography [126] accentuates ultrasound-guided puncture for the facilitation of puncture and possibly RAS reduction. Distal radial access is noted as more spasmogenic.

After gaining popularity and becoming the preferred access site for coronary interventions in most countries across the globe, radial access has attracted followers in other interventional areas, such as neurovascular procedures. A paper by Satti and Vance [127] brings up similar concepts as previously described in this analysis: patient education to minimize anxiety, local anesthetic cream application prior to the intervention, intravenous conscious sedation if called for, subcutaneous nitroglycerine and ultrasound-guided single-wall arterial puncture for increased first attempt success, and an intraarterial vasodilator cocktail consisting of nitroglycerine and verapamil. In addition to hydrophilic tapered sheath use, direct guiding catheter access is mentioned as a solution in case of small diameter radial artery. In case of important RAS, a previously unmentioned strategy is transient ulnar artery compression to deviate arterial flow toward the radial artery.

## 4. Discussion

RAS remains the main impeding factor in successful coronary interventions using the radial artery approach. The reported incidence is highly dependent on subjective diagnostic criteria; differences in the radial artery puncture experience, technique and materials are other confounding factors. A standardized definition could potentially help with future research on the matter, so that results between studies may be compared. Risk factors and predictive scores can help anticipate RAS and guide the operator toward more aggressive antispastic measures. Only some of these factors can be modified by patient intervention, such as mild patient sedation to quell anxiety.

Choosing smaller and hydrophilic materials (sheaths and catheters) and ultrasound-guided puncture are proven strategies that aid in RAS reduction. Tracking with balloons or small catheters are two highly effective and only mildly time-consuming methods that can be used for navigating a spastic radial artery.

Many small and medium-sized studies have addressed the intraarterial administration of spasmolytic drugs with calcium channel blockers and nitrates remaining the most cost-effective and efficient variants available. Although contraindications and side effects exist, no other medications have proven to be superior, and most operators use prophylactic cocktails containing nitroglycerine and verapamil upfront. It is interesting to note that one study argues that routine pharmacological prophylaxis carries no real benefit. Thus, an absolute indication for routine antispastics cannot be enforced, leaving room for larger randomized double-blinded studies aimed at settling this matter.

Topical and subcutaneous medication could prove to be useful adjuvant treatments, although only small studies have proven some benefit on RAS prevention when using anesthetic and vasodilating agents such as lidocaine and nitroglycerine. Mechanical compression and nerve block have evidence of effectiveness but are cumbersome to implement under real-world conditions. Extreme cases of unresolving spasm can be successfully tackled using lubricant solutions or thermal vasodilation.

From our own experience, consisting of more than 95% of coronary interventions performed using a right or left radial approach, we have found that a 5 Fr. TIG catheter allows one to successfully complete diagnostic coronary angiography in almost any patient, even females of very small stature, with minimal discomfort and without routine upfront vasodilator use. In case of difficulty advancing, it is best to stop and obtain an angiogram in order to plan accordingly; what one thinks is a spasm could be a loop. Possibly as a complementary to pharmacological treatment for RAS, pressure-mediated dilation is an interesting new method that warrants more research. A more pressing problem is coronary angioplasty when 6 or 7 Fr. guiding catheters are needed, but even these can successfully and effortlessly be advanced using the BAT technique, even if you are the lone operator and only have one set of hands. Anxiety quelling measures, in addition to medication, such as talking to the patient and music, often seem to help, although no trials on this exist. Future development may lead to the better screening of patients at risk of RAS and better understanding of the best measures to be taken, but with no current standardization, operator experience will probably continue to play a key role in securing best outcomes, with intraarterial nitroglycerine and verapamil the most cost effective, time efficient and widely available tools in spasm management.

## 5. Conclusions

The use of transradial access (TRA) for coronary artery interventions has several benefits, like less bleeding complications, early patient ambulation and reduced long-term costs, but radial artery spasm (RAS) is a frequent complication. The definition of RAS remains unstandardized, but a few predisposing factors may be considered to assess predictive risk. Prevention and treatment methods, from mechanical ones to intravenous or intraarterial vasodilator agents, topical and subcutaneous agents, mechanical compression, or even nerve block or sedation may be of great interest, but the future development and better screening of patients at risk of RAS may play a key role in standardization and spasm management.

## 6. Limitations

Heterogenous definitions are based often on subjective factors, which are highly operator and patient dependent, causing a high variability in reported spasm rates and making direct study comparison difficult. Small sample sizes in most of the analyzed studies means that they might be underpowered to detect useful interventions. Population differences, such as between Asian and Caucasian patients, in relation to body size for example, could also confound investigation results. Larger double-blinded studies with objective spasm definition criteria could shed more light on best practices in dealing with RAS.

## Figures and Tables

**Table 1:** Frequently cited clinical factors that operators use to define RAS (if two out of five present).

Clinical Factors Used to Define RAS
1. Patient reported presence of continuous forearm pain.
2. Patient reported forearm pain only during catheter manipulation.
3. Patient reported forearm pain during sheath insertion or retrieval.
4. Firm grip of the catheters during manipulation.
5. Augmented resistance to sheath retrieval.
